# Aqua­bis­(1-methyl-1*H*-imidazole-κ*N* ^3^)bis­(nitrato-κ*O*)copper(II)

**DOI:** 10.1107/S1600536810047525

**Published:** 2010-11-24

**Authors:** Run-Qiang Zhu

**Affiliations:** a Ordered Matter Science Research Center, College of Chemistry and Chemical Engineering, Southeast University, Nanjing 211189, People’s Republic of China

## Abstract

The title complex mol­ecule, [Cu(NO_3_)_2_(C_4_H_6_N_2_)_2_(H_2_O)], has crystallographically imposed twofold symmetry. The Cu^II^ atom displays a distorted square-pyramidal CuN_2_O_3_ coordination geometry. In the crystal, inter­molecular O—H⋯O hydrogen bonds between the coordinated water mol­ecule and the nitrate anions form chains parallel to the *c* axis.

## Related literature

The title compound was studied as part of our work to obtain potential ferroelectric phase-change materials. For general background to ferroelectric metal-organic frameworks, see: Fu *et al.* (2009 ▶); Ye *et al.* (2006 ▶); Zhang *et al.* (2008 ▶, 2010 ▶).
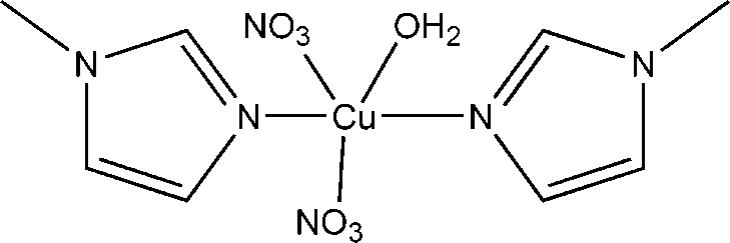


## Experimental

### 

#### Crystal data

[Cu(NO_3_)_2_(C_4_H_6_N_2_)_2_(H_2_O)]
*M* *_r_* = 369.78
Monoclinic, 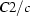

*a* = 11.864 (2) Å
*b* = 12.242 (2) Å
*c* = 10.509 (2) Å
β = 93.98 (3)°
*V* = 1522.6 (5) Å^3^
*Z* = 4
Mo *K*α radiation
μ = 1.48 mm^−1^
*T* = 293 K
0.30 × 0.25 × 0.20 mm

#### Data collection

Rigaku SCXmini diffractometer
Absorption correction: multi-scan (*CrystalClear*; Rigaku, 2005 ▶) *T*_min_ = 0.640, *T*_max_ = 0.740
7712 measured reflections
1742 independent reflections
1608 reflections with *I* > 2σ(*I*)
*R*_int_ = 0.031

#### Refinement

*R*[*F* ^2^ > 2σ(*F* ^2^)] = 0.033
*wR*(*F* ^2^) = 0.088
*S* = 1.14
1742 reflections
102 parameters
H-atom parameters constrained
Δρ_max_ = 0.45 e Å^−3^
Δρ_min_ = −0.37 e Å^−3^

### 

Data collection: *CrystalClear* (Rigaku, 2005 ▶); cell refinement: *CrystalClear*; data reduction: *CrystalClear*; program(s) used to solve structure: *SHELXS97* (Sheldrick, 2008 ▶); program(s) used to refine structure: *SHELXL97* (Sheldrick, 2008 ▶); molecular graphics: *SHELXTL* (Sheldrick, 2008 ▶); software used to prepare material for publication: *SHELXL97*.

## Supplementary Material

Crystal structure: contains datablocks I, global. DOI: 10.1107/S1600536810047525/rz2515sup1.cif

Structure factors: contains datablocks I. DOI: 10.1107/S1600536810047525/rz2515Isup2.hkl

Additional supplementary materials:  crystallographic information; 3D view; checkCIF report

## Figures and Tables

**Table 1:** Hydrogen-bond geometry (Å, °)

*D*—H⋯*A*	*D*—H	H⋯*A*	*D*⋯*A*	*D*—H⋯*A*
O1—H1*B*⋯O3^i^	0.85	2.48	2.941 (3)	115
O1—H1*C*⋯O3^ii^	0.85	2.48	2.941 (3)	115

Symmetry codes: (i) 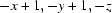; (ii) 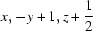.

**Fig. 1.**
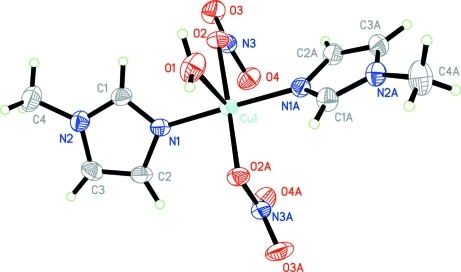
The molecular structure of the title compound, showing the atomic numbering scheme. Displacement ellipsoids are drawn at the 30% probability level. Symmetry code: (A) 1-x, y. 1/2-z.

**Fig. 2.**
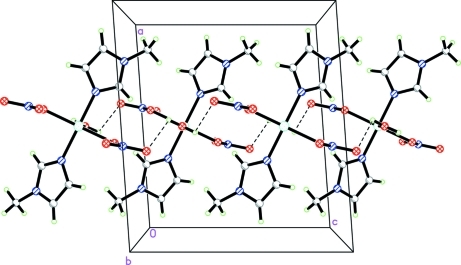
Packing diagram of the title compound showing the stacking of the molecules along the c axis. Dashed lines indicate hydrogen bonds.

## supplementary crystallographic information

### Comment

Dielectric constant measurements of compounds as a function of temperature is the basic method to find potential ferroelectric phase change materials (Fu *et al.*, 2009; Ye *et al.*, 2006; Zhang *et al.*, 2008; Zhang *et al.*, 2010). Unfortunately, the study carried out on the title compound indicated that the permittivity is temperature-independent, suggesting that there may be no dielectric disuniformity between 80 K to 350 K (m.p. 393–381 K). In this report the crystal structure of the title compound is reported.

The title complex molecules has crystallographically imposed twofold symmetry (Fig. 1). The copper(II) metal centre is five-coordinated in a distorted square-planar geometry by two nitrogen atoms from two 1-methyl-1*H*-imidazole ligands and two oxygen atoms from two NO_3_^-^ defining the basal plane, and a coordinated water at the apex. The Cu–N and Cu–O bond lengths are not exceptional. In the crystal packing, intermolecular O—H···O hydrogen bonds (Table 1) between the coordinate water molecules and nitrate ions form chains along the *c* axis (Fig. 2).

### Experimental

An aqueous solution of 1-methyl-1*H*-imidazole (1.64 g, 20 mmol) and H_2_SO_4_ (0.98 g, 10 mmol) was treated with CuSO_4_ (2.5 g, 10 mmol). After the mixture was churned for a few minutes, Ba(NO_2_)_2_ (5 g, 20 mmol) was added to give a blue solution. Slow evaporation of the resulting solution yielded blue crystals after a few days.

### Refinement

All H atoms were placed in geometrically idealized positions and constrained to ride on their parent atoms with C—H = 0.93–0.96 Å, O—H = 0.85 Å, and with *U*_iso_(H) = 1.2 *U*_iso_(C, O) or 1.5 *U*_iso_(C) for methyl H atoms.

### Crystal data

**Table:**

[Cu(NO_3_)_2_(C_4_H_6_N_2_)_2_(H_2_O)]	*F*(000) = 756
*M**_r_* = 369.78	*D*_x_ = 1.613 Mg m^−^^3^
Monoclinic, *C*2/*c*	Mo *K*α radiation, λ = 0.71073 Å
Hall symbol: -C 2yc	Cell parameters from 3705 reflections
*a* = 11.864 (2) Å	θ = 3.0–27.5°
*b* = 12.242 (2) Å	µ = 1.48 mm^−^^1^
*c* = 10.509 (2) Å	*T* = 293 K
β = 93.98 (3)°	Block, blue
*V* = 1522.6 (5) Å^3^	0.30 × 0.25 × 0.20 mm
*Z* = 4	

### Data collection

**Table:**

Rigaku SCXmini diffractometer	1742 independent reflections
Radiation source: fine-focus sealed tube	1608 reflections with *I* > 2σ(*I*)
graphite	*R*_int_ = 0.031
CCD_Profile_fitting scans	θ_max_ = 27.5°, θ_min_ = 3.0°
Absorption correction: multi-scan (*CrystalClear*; Rigaku, 2005)	*h* = −15→15
*T*_min_ = 0.640, *T*_max_ = 0.740	*k* = −15→15
7712 measured reflections	*l* = −13→13

### Refinement

**Table:**

Refinement on *F*^2^	Primary atom site location: structure-invariant direct methods
Least-squares matrix: full	Secondary atom site location: difference Fourier map
*R*[*F*^2^ > 2σ(*F*^2^)] = 0.033	Hydrogen site location: inferred from neighbouring sites
*wR*(*F*^2^) = 0.088	H-atom parameters constrained
*S* = 1.14	*w* = 1/[σ^2^(*F*_o_^2^) + (0.0453*P*)^2^ + 0.8389*P*] where *P* = (*F*_o_^2^ + 2*F*_c_^2^)/3
1742 reflections	(Δ/σ)_max_ < 0.001
102 parameters	Δρ_max_ = 0.45 e Å^−^^3^
0 restraints	Δρ_min_ = −0.37 e Å^−^^3^

### Special details

**Table:**

Geometry. All e.s.d.'s (except the e.s.d. in the dihedral angle between two l.s. planes) are estimated using the full covariance matrix. The cell e.s.d.'s are taken into account individually in the estimation of e.s.d.'s in distances, angles and torsion angles; correlations between e.s.d.'s in cell parameters are only used when they are defined by crystal symmetry. An approximate (isotropic) treatment of cell e.s.d.'s is used for estimating e.s.d.'s involving l.s. planes.
Refinement. Refinement of *F*^2^ against ALL reflections. The weighted *R*-factor *wR* and goodness of fit *S* are based on *F*^2^, conventional *R*-factors *R* are based on *F*, with *F* set to zero for negative *F*^2^. The threshold expression of *F*^2^ > σ(*F*^2^) is used only for calculating *R*-factors(gt) *etc*. and is not relevant to the choice of reflections for refinement. *R*-factors based on *F*^2^ are statistically about twice as large as those based on *F*, and *R*- factors based on ALL data will be even larger.

### Fractional atomic coordinates and isotropic or equivalent isotropic displacement parameters (Å^2^)

**Table:**

	*x*	*y*	*z*	*U*_iso_*/*U*_eq_	Occ. (<1)
C1	0.3268 (2)	0.4147 (2)	0.0458 (2)	0.0496 (5)	
H1A	0.3799	0.4495	−0.0012	0.060*	
C2	0.2500 (2)	0.3234 (2)	0.1908 (2)	0.0505 (5)	
H2	0.2402	0.2830	0.2641	0.061*	
C3	0.1669 (2)	0.3521 (2)	0.1028 (3)	0.0543 (6)	
H3A	0.0905	0.3354	0.1042	0.065*	
C4	0.1617 (3)	0.4595 (3)	−0.1034 (3)	0.0765 (9)	
H4A	0.1108	0.5157	−0.0797	0.115*	
H4B	0.1203	0.4044	−0.1519	0.115*	
H4C	0.2178	0.4906	−0.1541	0.115*	
Cu1	0.5000	0.36656 (3)	0.2500	0.03738 (14)	
N1	0.35145 (16)	0.36341 (14)	0.15477 (17)	0.0427 (4)	
N2	0.21687 (16)	0.41042 (16)	0.01160 (19)	0.0493 (4)	
N3	0.58487 (15)	0.27708 (16)	0.03266 (17)	0.0462 (4)	
O1	0.5000	0.5610 (2)	0.2500	0.0700 (8)	
H1B	0.5267	0.5958	0.1888	0.084*	0.50
H1C	0.4733	0.5958	0.3112	0.084*	0.50
O2	0.57326 (14)	0.37175 (12)	0.08207 (15)	0.0473 (4)	
O3	0.60599 (18)	0.27180 (18)	−0.08000 (16)	0.0715 (6)	
O4	0.57213 (17)	0.19602 (15)	0.09852 (17)	0.0650 (5)	

### Atomic displacement parameters (Å^2^)

**Table:**

	*U*^11^	*U*^22^	*U*^33^	*U*^12^	*U*^13^	*U*^23^
C1	0.0505 (12)	0.0489 (12)	0.0495 (12)	−0.0099 (10)	0.0038 (10)	0.0080 (10)
C2	0.0491 (12)	0.0617 (14)	0.0414 (11)	−0.0145 (11)	0.0085 (9)	0.0018 (10)
C3	0.0433 (12)	0.0639 (15)	0.0559 (14)	−0.0101 (10)	0.0051 (10)	−0.0042 (11)
C4	0.0762 (19)	0.0697 (18)	0.080 (2)	−0.0042 (15)	−0.0213 (16)	0.0228 (15)
Cu1	0.0392 (2)	0.0413 (2)	0.0326 (2)	0.000	0.00887 (13)	0.000
N1	0.0436 (9)	0.0461 (10)	0.0391 (9)	−0.0060 (7)	0.0072 (7)	−0.0004 (7)
N2	0.0522 (11)	0.0427 (10)	0.0520 (11)	−0.0038 (8)	−0.0038 (9)	0.0025 (8)
N3	0.0437 (9)	0.0580 (11)	0.0371 (9)	0.0025 (8)	0.0052 (7)	−0.0051 (8)
O1	0.096 (2)	0.0467 (14)	0.0651 (16)	0.000	−0.0127 (15)	0.000
O2	0.0532 (9)	0.0481 (9)	0.0422 (8)	−0.0034 (6)	0.0152 (7)	−0.0010 (6)
O3	0.0866 (14)	0.0926 (15)	0.0375 (9)	0.0187 (11)	0.0187 (8)	−0.0079 (9)
O4	0.0866 (13)	0.0497 (10)	0.0582 (10)	−0.0069 (9)	0.0013 (9)	0.0026 (8)

### Geometric parameters (Å, °)

**Table:**

C1—N1	1.321 (3)	C4—H4C	0.9600
C1—N2	1.330 (3)	Cu1—N1^i^	1.9658 (19)
C1—H1A	0.9300	Cu1—N1	1.9658 (19)
C2—C3	1.350 (3)	Cu1—O2^i^	2.0216 (16)
C2—N1	1.377 (3)	Cu1—O2	2.0216 (16)
C2—H2	0.9300	Cu1—O1	2.381 (3)
C3—N2	1.363 (3)	N3—O4	1.225 (3)
C3—H3A	0.9300	N3—O3	1.229 (2)
C4—N2	1.463 (3)	N3—O2	1.281 (2)
C4—H4A	0.9600	O1—H1B	0.8500
C4—H4B	0.9600	O1—H1C	0.8500
			
N1—C1—N2	111.7 (2)	N1—Cu1—O2	88.90 (8)
N1—C1—H1A	124.2	O2^i^—Cu1—O2	176.40 (9)
N2—C1—H1A	124.2	N1^i^—Cu1—O1	91.12 (5)
C3—C2—N1	109.3 (2)	N1—Cu1—O1	91.12 (5)
C3—C2—H2	125.4	O2^i^—Cu1—O1	88.20 (4)
N1—C2—H2	125.4	O2—Cu1—O1	88.20 (4)
C2—C3—N2	106.6 (2)	C1—N1—C2	105.21 (19)
C2—C3—H3A	126.7	C1—N1—Cu1	124.65 (16)
N2—C3—H3A	126.7	C2—N1—Cu1	129.59 (15)
N2—C4—H4A	109.5	C1—N2—C3	107.3 (2)
N2—C4—H4B	109.5	C1—N2—C4	125.5 (2)
H4A—C4—H4B	109.5	C3—N2—C4	127.2 (2)
N2—C4—H4C	109.5	O4—N3—O3	122.9 (2)
H4A—C4—H4C	109.5	O4—N3—O2	118.86 (17)
H4B—C4—H4C	109.5	O3—N3—O2	118.2 (2)
N1^i^—Cu1—N1	177.76 (10)	Cu1—O1—H1B	120.0
N1^i^—Cu1—O2^i^	88.90 (8)	Cu1—O1—H1C	120.0
N1—Cu1—O2^i^	91.17 (8)	H1B—O1—H1C	120.0
N1^i^—Cu1—O2	91.17 (8)	N3—O2—Cu1	112.97 (12)

Symmetry codes: (i) −*x*+1, *y*, −*z*+1/2.

### Hydrogen-bond geometry (Å, °)

**Table:**

*D*—H···*A*	*D*—H	H···*A*	*D*···*A*	*D*—H···*A*
O1—H1B···O3^ii^	0.85	2.48	2.941 (3)	115
O1—H1C···O3^iii^	0.85	2.48	2.941 (3)	115

Symmetry codes: (ii) −*x*+1, −*y*+1, −*z*; (iii) *x*, −*y*+1, *z*+1/2.
